# Combined Treatments with Photodynamic Therapy for Non-Melanoma Skin Cancer

**DOI:** 10.3390/ijms161025912

**Published:** 2015-10-28

**Authors:** Silvia Rocío Lucena, Nerea Salazar, Tamara Gracia-Cazaña, Alicia Zamarrón, Salvador González, Ángeles Juarranz, Yolanda Gilaberte

**Affiliations:** 1 Department of Biology, Faculty of Sciences, Autónoma University, 28049 Madrid, Spain; E-Mails: silvialucenablas@gmail.com (S.R.L.); nerea.salazar@estudiante.uam.es (N.S.); aliszm@gmail.com (A.Z.); angeles.juarranz@uam.es (A.J.); 2 Unit of Dermatology, Barbastro Hospital, 22300 Barbastro, Spain; E-Mail: tamara_gracia@hotmail.com; 3 Aragón Health Sciences Institute, 50009 Zaragoza, Spain; 4 Department of Medicine and Medical Divisions, University of Alcalá, 28801 Madrid, Spain; E-Mail: gonzals6@mskcc.org; 5 Memorial Sloan Kettering Cancer Center, New York, NY 10022, USA; 6 Unit of Dermatology, San Jorge Hospital, 22005 Huesca, Spain

**Keywords:** photodynamic therapy, methyl-aminolevulinic acid, aminolevulinic acid, actinic keratosis, basal cell carcinoma, Bowen disease, squamous cell carcinoma, tumor resistance

## Abstract

Non-melanoma skin cancer (NMSC) is the most common form of cancer in the Caucasian population. Among NMSC types, basal cell carcinoma (BCC) has the highest incidence and squamous cell carcinoma (SCC) is less common although it can metastasize, accounting for the majority of NMSC-related deaths. Treatment options for NMSC include both surgical and non-surgical modalities. Even though surgical approaches are most commonly used to treat these lesions, Photodynamic Therapy (PDT) has the advantage of being a non-invasive option, and capable of field treatment, providing optimum cosmetic outcomes. Numerous clinical research studies have shown the efficacy of PDT for treating pre-malignant and malignant NMSC. However, resistant or recurrent tumors appear and sometimes become more aggressive. In this sense, the enhancement of PDT effectiveness by combining it with other therapeutic modalities has become an interesting field in NMSC research. Depending on the characteristics and the type of tumor, PDT can be applied in combination with immunomodulatory (Imiquimod) and chemotherapeutic (5-fluorouracil, methotrexate, diclofenac, or ingenol mebutate) agents, inhibitors of some molecules implicated in the carcinogenic process (COX2 or MAPK), surgical techniques, or even radiotherapy. These new strategies open the way to a wider improvement of the prevention and eradication of skin cancer.

## 1. Skin Cancer

Skin cancer is a main common form of cancer, with non-melanoma types (NMSC), basal cell carcinoma (BCC) and squamous cell carcinoma (SCC), being the most frequent types [1,2]. Although death from NMSC is rare, treatment of NMSC results in a considerable burden on the health-care system [3,4,5].

One of the most important etiologic factors is sun exposure, particularly, ultraviolet radiation (UVR) and especially ultraviolet B (UVB). In fact, exposure to UVR is associated with approximately 90% of NMSCs. This is particularly applicable to people who have lighter complexion (skin types I–III) [6] and some predisposing genetic factors [7].

**Figure 1:**
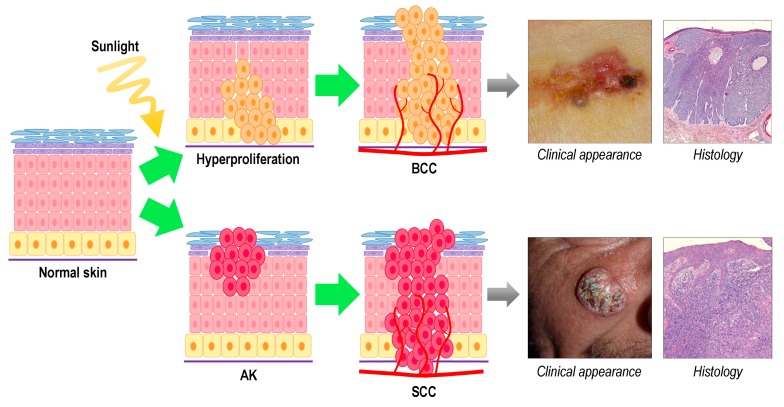
Formation process, clinical and histological appearance of basal cell carcinoma and squamous cell carcinoma.

BCC is the most common cancer in fair skinned individuals and its incidence is still rising [6]. The incidence is also increasing among younger people (<40 years), however the average age of first diagnosis is 60. Metastasis is unusual (incidence of 0.0028%–0.55%), however, the invasive growth pattern can destroy vital structures [8]. It mainly appears in the chronically photoexposed skin, even though superficial BCC is also frequent on the trunk. Other risk factors include skin phototype, immunosuppression, and hereditary disorders such as nevoid basal cell carcinoma syndrome (Gorlin-Goltz syndrome) and xeroderma pigmentosum. Recently, it has been proposed that 90% of superficial BCC evolve from interfollicular epidermal basal stem cells, whereas the rest of types evolve from hair follicle (Figure 1) [9].

According to both clinical and histological criteria, the most common subtypes of BCC are nodular, infiltrative (sclerodermiform and micronodular) and multicentric-superficial. Aggressive ulcerative and destructive forms and pigmented BCCs are other variants [6,8].

SCC derives from the keratinocytes of the spinous layer of the epidermis (Figure 1). Although it has a lower frequency in relation to BCC, SCC is more aggressive and has a major capacity of metastasis, thus being responsible for most deaths associated with NMSC. It also appears in photoexposed areas such as the head, neck and extremities and its incidence increases with age [4,10,11,12,13]. Most cases of SCC derive from chronic UV-radiation exposure being the premalignant lesion actinic keratosis (AK). Otherwise, Bowen Disease (BD) or *SCC in situ*, could evolve into an invasive SCC (iSCC) in some cases. In contrast to BD, in iSCC atypical keratinocytes are present beyond the basement membrane, in the dermis or deeper, and it could metastasize. On the other hand, SCC is the most common malignant tumor in transplant patients, who present multiple lesions and with more aggressive behavior than in the general population [14].

AK is a cutaneous lesion and develops mostly in fair skinned patients susceptible to solar damage. Its frequency is directly related to cumulative exposure of UV, and therefore, usually develops on sun-exposed areas (face, forearms, upper back and legs). AK is often multiple and usually found in an area of “cancerization field” that defines the skin around AK, which provides the basis for clonal expansion of genetically altered neoplastic cells [4]. SCC and AK essentially represent the same disease process but at different stages of evolution [15,16]. It is estimated that between 5% and 10% of AK progress to BD or SCC [6,17].

## 2. Photodynamic Therapy for Non-Melanoma Skin Cancer

There are several classical therapies for the different types of NMSC. In general, the most widely extended is surgery but other treatments are: (1) for BCC curettage and electrodessication, radiotherapy, cryotherapy, photodynamic therapy (PDT), laser, topical (imiquimod and 5-fluorouracil), and systemic treatments (vismodegib and sonidegib [18]; and (2) for SCC cryosurgery, electrosurgery and radiotherapy; topical treatments such as 5-fluorouracil and imiquimod or PDT are only recommended for SCC *in situ*. Regarding AK cryotherapy, PDT, topical 5-fluorouracil (5%, 1%, 0.5%), imiquimod (5%, 3.75%), ingenol mebutate (0.05%, 0.015%), diclofenac, topical retinoids, chemical peels, electrosurgery or lasers are used to treat isolated or multiple lesions [19].

Among all the alternative treatments used for non-melanoma skin cancer, we highlight PDT not only because of its high efficacy (strength of recommendation A, quality of evidence I for AK, BD and BCC) but also for its good cosmetic outcome [1,6,20,21,22,23,24,25]. As it has those advantages, PDT treatment has been extensively developed as a new modality and an alternative to classical ones such as surgery, radiation or chemotherapy, being particularly suitable for treating multiple lesions and field cancerization [26]. PDT is more beneficial in those patients in which size, site or number of lesions limit the efficacy and/or acceptability of conventional therapies [27].

This treatment is comparatively non-invasive and is based on the interaction of three elements: a photosensitizing compound, light of an adequate wavelength and oxygen. When the photosensitizer (PS) is activated by light, it triggers a photochemical reaction by which singlet oxygen (^1^O_2_) and other reactive oxygen species (ROS) are produced and selectively kill cancer cells [27,28,29,30]. Anti-tumoral effects of PDT derive from three principal mechanisms: direct cytotoxicity on cancer cells, indirect effects consequence of damage to the tumor vasculature, and the activation of the immune response.

The principal compounds used in cutaneous oncology are: 5 aminolevulinic acid (ALA, Levulan^®^ (Wilmington, DE, USA) and Ameluz^®^ (Leverkusen, Germany) and its methylated derivate (MAL, Metvix^®^ (Alby sur Cheran, France)) [27,31]. MAL is approved in Europe for the treatment of AK, BD, and for superficial and nodular (less than 2 mm of deepness) BCC, whereas ALA is only indicated for AK. ALA is a precursor of intermediate porphyrins of heme group biosynthesis, specifically of the photoactive compound Protoporphyrin IX (PpIX). ALA administration results in the production and selective accumulation of the PS PpIX in cancer cells. There are also some studies about the use of PDT for other skin cancer types such as lymphomas [26].

It has been reported that PDT exerts an anti-tumor immunological effect. This sometimes could be associated with an intense inflammatory response, characterized by an increase of cytokines and accumulation of leukocytes in targeted tumor areas, favoring tumor destruction. On the other hand, Evangelou *et al.* [32] revealed that MAL-PDT of BCC reduces local epidermal Langerhans cells. Thus, this suppression of the skin immune response might potentially result in a less effective antitumor response.

### 2.1. Limitations of Photodynamic Therapy (PDT)

Among the benefits of MAL-PDT are its high rates of complete response (CR); in BCC, 91% at three months and 76% at five years of follow up, and in BD, 86% to 93% at three months and 68% to 71% at 24 months [33]. Other advantages include the possibility of combining PDT with other therapies and repeat the process as needed with an excellent cosmetic result and greater satisfaction of the patient. However its limitations are, pain during illumination [34,35], the penetration of the photosensitizer and light, and the possibility of tumor resistance, occasionally found in those refractory to treatment [36,37]. Next, we describe the factors that may limit the response to PDT.

Pain is the most common adverse effect in PDT and it is one of the limiting factors in some patients. Such pain seems due to the combination of heat and intense nerve stimulation. While between 60% and 80% of patients consider the pain light to moderate, 20% describe it as intense [34]. It has been observed that this technique is less painful when using MAL than ALA, being pre-treatment PpIX fluorescence and lesion diameter associated with pain [36].

The intensity of pain is related to the area of treatment, the amount of pain receptor nerves at the tumor site (more in head, hands and perineum), male patients, phototype and in the second cycle of PDT treatment [33,37]. Furthermore, it has been hypothesized that pain could play a role in the pathogenesis of blood pressure increase after MAL-PDT, including acute postoperative hypertension and hypertensive crisis [38].

Tumoral thickness is the best-studied limiting factor of PDT for BCC. McKay *et al.* [39] described those tumors thicker than 0.4 mm had more risk of recurrence and, on the other hand, PDT is not recommended for nodular BCC deeper than 2 mm [24]. However, for superficial BCC no association was found between thickness and adnexal extension and treatment failure [40]. To reduce the impact of this limitation, different techniques have been used to diminish the tumor size or to increase the penetration of the photosensitizer; Christensen *et al.* [41] showed that a deep curettage which, reduced the tumor thickness by 50%, improved the outcome in patients following PDT in BCC with mean thickness of 2.3 mm (CR at three months of 93%).

Some histological subtypes of BCC are more resistant to PDT than others. Pigmented BCC responds worse due to light absorption by melanin [42]; the high density of collagen and the cords of cells infiltrating the connective tissue limits the penetration of PS in the morphoeic and infiltrative variants of BCC, respectively [42]. For these reasons, PDT is not indicated in these subtypes of tumor.

Hyperkeratosis is one of the principal inconveniences for the photosensitizer penetration. The use of keratolytic agents or curettage before PDT is mandatory for a successful PDT [28].

The size of the tumor is a limiting factor for some authors. Madan *et al.* found that giant superficial BCC [43] or those tumors over 4 cm in diameter respond worse to PDT [44,45]. However, there are more studies supporting that tumor diameter does not affect the outcome [46,47,48,49,50]. In BD, clinical guidelines consider PDT as a first line treatment, especially in big and multiple lesions [49,51,52]. Whereas some authors conclude that tumor diameter is not a predictor of final response, although it has been associated with tumor relapse [53], others found it a negative prognostic factor [54].

The site of the lesions could have a strong influence over PDT response. BCC located on the face/head has lower complete response rates compared to those on the trunk/neck region (at 24 months, 54% *vs.* 88%) [48]. If the tumor is present in the H zone of the face, regardless of size, the response is also reduced; considering also that this is a risk zone for BCC, PDT is not indicated in this area [48]. Regarding AK, lesions present on the limbs are more resistant to MAL-PDT than those located on the face/scalp [55,56]. Modern guidelines consider BD located on fingers as a limitation factor for the use of PDT [57].

Another factor that may limit the effectiveness of PDT can be patient’s age, being more effective in younger patients [58]. Previous history of radiotherapy in the area has been also associated with less response of BCC [59].

### 2.2. Resistance to PDT

Resistance to traditional anti-cancer therapies (chemotherapy and radiotherapy) is the main cause of their failure, leading to tumor progression and poor clinical prognoses. Chemotherapy success is associated with factors related to the access of drugs to subcellular sites, cell-cycle kinetics and/or mutations [60,61,62,63,64]. Concerning PDT, efficacy mainly depends on the selective uptake of the photosensitizer by tumoral cells, oxygen levels, and irradiation dose. Although it is not well documented, PDT can also induce tumor cell resistance in patients [65,66,67,68].

As a response to PTD, some genes clearly addressed to cell death and others to its protection are expressed. The activation of these oxidative stress genes could contribute to a poor response to the treatment [69,70]. Some of the genes activated after PDT are nuclear factor K (NF-κB), mitogen-activated protein kinase (MAPK), protein kinase B (PKB/Akt), phosphoinositide-3 kinase (PI3K) and cyclooxygenase 2 (COX-2). The signaling mediated by MAPK is implicated in numerous physiological processes in response to stress, like proliferation and cell death [71]. Otherwise, COX, which participates in prostaglandin synthesis (PGE), is related to the immunomodulatory responses development. The isoform COX-2 is implicated in inflammatory processes and in cancer through the synthesis of PEG2 overexpressed in premalignant and malignant situations of epithelial lesions. The expression of PEG2 is related to the activation of survival, proliferation and apoptosis inhibition signals mediated by MAPKs, PI3K/Akt. In the same way, the overexpression of COX-2 in NMSC has been described and can be considered as an early marker of actinic damage in carcinogenesis [72,73]. On the other hand, MAL-PDT, as has been said before, reduces local epidermal Langerhans cells [31].

The combination of diverse therapeutic modalities is one of the new strategies to enhance oncologic treatments. In order to overcome the limitations of PDT and to prevent the development of resistance, the application of PDT combined with coadjutant therapies, without increasing the toxicity for the patient, is being widely investigated.

## 3. Combined Treatments with PDT in NMSC

**Figure 2:**
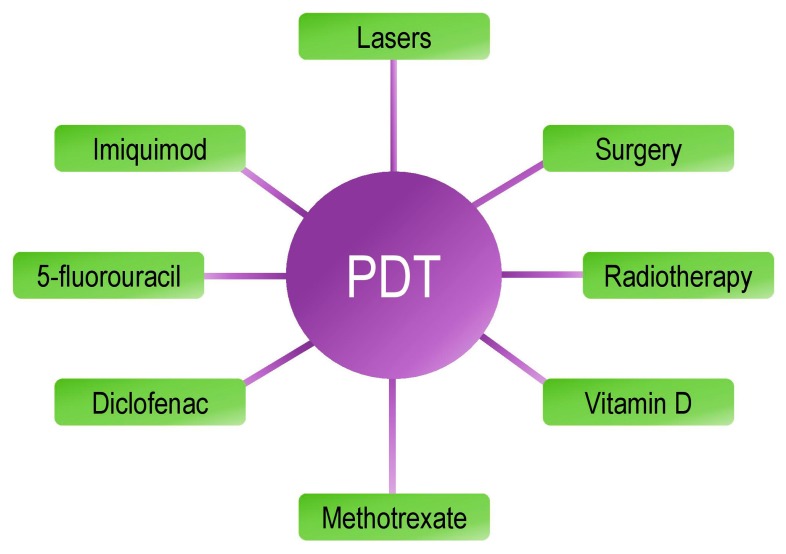
Treatments and procedures that have been combined with photodynamic therapy to treat non-melanoma skin cancer.

NMSCs are treated mainly by surgery, but there are other therapies less invasive such us 5-fluorouracil, the immunomodulator imiquimod, the nonsteroidal anti-inflammatory diclofenac and other agents such as ingenol mebutate. However, occasionally after treatment, recurrence appears, so a better understanding of its causes would be essential to develop more effective therapies [74]. The combination of various therapeutic modalities is an interesting strategy in cancer management. Next, we will review the different treatments used in combination with PDT for NMSC (Figure 2).

### 3.1. Actinic Keratosis

Table 1 summarizes AK studies combining PDT with other treatments. 5-fluorouracil (5-FU) induces cell cycle arrest and apoptosis and is used in the treatment of AK and superficial BCC twice-daily for two or more weeks [75]. In a study with 15 patients with facial AK applied 5-FU nightly for five days and underwent ALA-PDT on the sixth day. At one month and one year post-treatment, 90% of treated AK had resolved [76]. Martin reported in 2011 [77] that the treatment with sequential 5-FU and ALA-PDT in three old men with AK, when compared with ALA-PDT alone, was more effective, minimized the recurrence of areas of field cancerization and improves the appearance of the skin.

Imiquimod 5% cream stimulates the innate immune response and is applied between twice and five times a week for 6 to 16 weeks, depending on whether it is being used for AK, or superficial BCC [75]. In a study from 2009 [78] it was demonstrated that ALA-PDT followed by imiquimod (twice a week for 16 weeks) was more effective than PDT alone. At month 12, the median lesion reduction was 89.9% in patients who were treated with combined therapy *vs.* 74.5% of PDT alone. Serra-Guillén *et al.* [79] also demonstrated that PDT and imiquimod was more efficacious that PDT alone, but more patients were satisfied with PDT than with the other two modalities. In a more recent study, three patients received one session of PDT plus imiquimod three days per week for one month, and those without complete response underwent a second four-week course. Two patients showed complete clinical clearance of AK [80].

Diclofenac is a non-steroidal anti-inflammatory drug that reduces the production of prostaglandins by inhibiting inducible COX-2. The approved treatment regimen for AK consists of twice-daily application for 60 to 90 days [75]. Akita *et al.* [81] showed a considerable expression of COX-2 in AK (15 of 25, 60%) and BD (13 of 17, 76%). In contrast, only one of 33 (3%) BCC was a COX-2 high expresser. Bagazgoitia *et al.* [82] also observed that COX-2 is overexpressed in AK and BD, and its expression could be correlated with the PDT response. In a study with 10 patients, the pretreatment with diclofenac plus ALA-PDT reduced the number of lesions compared to PDT alone after 12 months [83].

Ingenol mebutate appears to have multiple mechanisms of action, including direct cell death and a complex inflammatory response [75]. Berman *et al.* [84] carried out a study with 24 patients that were divided in two treatments with ALA-PDT, 1 ALA-PDT treatment and one course of ingenol mebutate daily for three consecutive days, or one course of ingenol mebutate alone. Subjects in the two ALA-PDT treatment group had a 97.5% mean reduction from the number of baseline AK; ALA-PDT plus ingenol mebutate gel reached 86.7% mean reduction; while subjects in the ingenol mebutate group had a 91.7% mean reduction.

Fractional laser produces small columns of thermal injury to the skin, which are known as microthermal zones (MTZs). These MTZs vary from one device to another. Some are nonablative dermal injuries only, whereas others are associated with ablative changes in the skin, causing both epidermal and dermal injury patterns. In this sense, Togsverd-Bo *et al.* [85] compared ablative fractional laser (AFL)-assisted daylight photodynamic therapy (AFL-dPDT) with daylight PDT (dPDT), conventional PDT (cPDT) and AFL alone (AFL) in field treatment of AK in organ-transplant recipients. Sixteen patients with a total of 542 AK were treated. Three months later, complete response rates were 74% using AFL-dPDT, 46% dPDT, 50% cPDT and 5% AFL.

**Table 1:** Clinical studies of combined treatments with photodynamic therapy for actinic keratoses.

PS (PDT)	Coadyuvant	*n* (Patients)	Results	Adverse Effects	Reference
ALA	5-fluorouracil	15	At 1 month and at 1 year post-PDT + 5-FU treatment, 90% of treated AKs had resolved in all but one patient.	Not reported	[76]
ALA	5-fluorouracil	3	PDT + 5-FU treatment is more effective, minimizes the recurrence of areas of field cancerization and improves the appearance of the skin, in comparison with PDT alone	Not reported	[77]
ALA	Imiquimod	3	At 7 and 11 months after PDT + imiquimod treatment, 2 patients showed complete clearance of AKs	Skin reactions such as severe erythema, scaling, and crusting	[80]
ALA	Imiquimod	25	At month 12, median lesion reduction was 89.9% after PDT + imiquimod and 74.5% after PDT	Severe local skin reactions in most participants: erythema, flaking, scaling and dryness	[78]
MAL	Imiquimod	105	Complete clinical response: 37.5% after PDT + imiquimod, 10% after PDT and 27% after imiquimod. Histological response (absence of AK): 84% after PDT + imiquimod, 55% after PDT and 79% after imiquimod. Complete clinicopathologic response: 34% after PDT + imiquimod, 10% after PDT and 27% after imiquimod	90% of patients who received PDT were very satisfied with treatment *versus* 61% of patients who received imiquimod	[79]
ALA	Diclofenac	10	At 12 months, significantly fewer AK were seen in the PDT + diclofenac group compared with PDT alone	Pain during PDT was greater in the PDT + Diclofenac group	[83]
ALA	Ingenol mebutate	24	Mean reduction of the number of Aks: 86.7% in PDT + ingenol group 97.5% in PDT group 91.7% in ingenol mebutate group	Not reported	[84]
MAL-PDT: conventional (cPDT) and daylight (dPDT)	Ablative Fractional Laser (AFL)	16	Complete response rates: 74% in AFL + dPDT group 46% in dPDT group 50% in cPDT group 5% in AFL group	Erythema and crusting were more intense following AFL-dPDT than dPDT or cPDT	[85]

ALA: aminolevulinic acid; MAL: methyl-aminolevulinate; PDT: photodynamic therapy; cPDT: conventional PDT; dPDT: daylight PDT; AFL: ablative gractional laser; AK: actinic keratosis; 5-FU: 5 fluorouracil.

### 3.2. Squamous Cell Carcinoma

Table 2 summarizes the clinical studies combining PDT with other therapies for SCC. Although BD is probably the tumor with better response to PDT, some can show resistance or recurrence after PDT. Lu *et al.* [86] evaluated 13 cases of BD treated with ALA-PDT (three times) after surgery and there was no recurrence in one year. In addition, Sotiriou *et al.* [87] evaluated the combination of PDT plus imiquimod. They treated a man with a two-year history of BD on his face with two PDT sessions followed by application of imiquimod and complete clinical and histological response was achieved.

For decades radiotherapy has been a treatment option for NMSC when other modalities failed or could not be used. Nevertheless, the side effects of ionizing radiation are well known and the amount of radiation applied is limited [88]. Nakano *et al.* [89] selected four patients with BD that did not show complete remission or showed recurrence after ALA-PDT. Then, they were again treated with ALA-PDT followed by radiotherapy; the combination therapy was repeated every two to three days for a total of four treatments. All lesions disappeared and recurrence was not detected during 14 months.

Finally, Cai *et al.* [90] evaluated the efficacy of ALA-PDT in combination with CO_2_ laser. Twenty-two lesions were randomized into two groups: 11 lesions were treated with ALA-PDT and CO_2_ laser and 11 lesions with CO_2_ laser alone. In the ALA-PDT  +  CO_2_ laser group, eight out of 11 BD lesions showed complete remission, and only one recurred. In the control group, seven lesions out of 11 completely cleared and five recurred.

Most studies about PDT plus other therapeutic agent in SCC have been done in cellular mice models, because PDT is not recommended for invasive SCC. However, Lu *et al.* [86] evaluated the efficacy of ALA-PDT plus excision in five cases of SCC. All cases were treated with topical ALA-PDT (three times) after surgery. There was no recurrence in six months after treatment but two cases did one year later.

There are several *in vitro* studies combining ALA-PDT with determined compounds. For example the combination ALA-PDT with Nimesulide (COX-2 inhibitor) was determined on two SCC cell lines, HSC-2 and HSC-4, with different COX-2 expression. Nimesulide had an inhibitory effect on HSC-2 (COX-2 high expresser), but not on HSC-4 (COX-2 non-expresser). ALA-PDT showed an inhibitory effect on both cell lines. The combination of PDT with Nimesulide provoked a significant synergistic effect on the inhibition of cellular growth, especially in HSC-2 [81]. Ishida *et al.* [91] found that HSC-5 cells, another human SCC cell line, pretreated with etretinate (a retinoid) were more susceptible to ALA-PDT by enhanced accumulation of intracellular PpIX.

Methotrexate (MTX) is a chemotherapeutic agent that inhibits cell proliferation and triggers cellular differentiation. Preincubation of human skin carcinoma cells SCC13 and normal keratinocytes HEK1 with MTX followed by incubation with ALA significantly enhanced the intracellular porphyrin levels in the cells and therefore, PDT was synergistically enhanced by MTX treatment. These effects were also described in SKH-1 hairless mice tumors previously treated systemically with MTX to ALA-PDT [92].

Vitamin D is a pro-hormone synthesized in the keratinocytes in a reaction catalyzed by UV radiation, and it can also be taken from the diet. In the skin, the active form of vitamin D can be completely produced by the keratinocytes and contribute to maintain the quiescence of differentiated phenotype and inhibit modified signaling pathways in NMSC such as Wnt/β-catenin and Hedgehog/Patched [93,94,95]. Cicarma *et al.* [96] demonstrated that a short course (24 h) of calcitriol pretreatment before MAL-PDT elevated the intracellular PpIX in A-431 cells. Furthermore, cell damage after exposure to blue light was significantly higher in calcitriol treated cells. Increased photoinactivation correlated with higher levels of PpIX in the calcitriol-pretreated cells. Likewise, Anand *et al.* [97] using mouse models of SCC reported that calcitriol administered prior to ALA increased the efficacy of PDT. Anand *et al.* [98] also tested a possible strategy to overcome the problem of hypercalcemia by substituting natural dietary vitamin D3 for calcitriol. Oral D3 supplementation enhanced PpIX levels, and cell death in subcutaneous A431 tumors mediated by PDT. These results support the brief use of oral administration of cholecalciferol as a safe neoadjuvant to ALA-PDT. In BCC and UVB-induced SCC mouse models, they identified an increase in tumor-specific accumulation of PpIX due to vitamin D preconditioning of up to six-fold *in vivo*. In addition, increased expression of proliferation (42-fold) and differentiation (145-fold) markers was identified in BCCs, all leading to superior tumor destruction (18.3-fold) with the combination of both treatments, compared to ALA-PDT [99].

Furthermore, in our previous works, one of the factors that seemed related to the lack of response to PDT was the overexpression of EGFR [100]. For this reason, association of PDT with an EGFR blocker may help to reduce treatment resistance [101]. Weyergang *et al.* [102] evaluated the combination of PDT plus EGFR targeting drugs in A-431 cells, an EGFR positive cell line of human epidermoid skin carcinoma. PDT and Tyrphostin revealed synergistic cytotoxicity, whereas Erlotinib or Cetuximab induced an antagonistic effect on cell survival. Neoadjuvant EGFR targeting therapies and PDT induced a synergistic inhibition of ERK as well as synergistic cytotoxicity only when the EGFR targeting monotherapies caused a prolonged ERK inhibition.

### 3.3. Basal Cell Carcinoma

Different treatments have been combined with PDT to increase its efficacy and overcome resistances (Table 3). Jeremic *et al.* [103] treated 10 BCC and eight SCC previous to surgical excision; four BCC demonstrated a complete response after an average of two PDT treatments, whereas the remaining lesions demonstrated a partial response after three PDT sessions with a maximum reduction of the tumor area of 88%. These tumors were then excised with clear histologic margins without recurrence in one year. The results suggest that for those NMSC without a complete response to PDT, neoadjuvant PDT can substantially reduce tumor burden, allowing for less morbid surgical excisions with histologically clear margins. In another study, Torres *et al.* [104] showed that two PDT sessions, one week apart, reduced in 30%–50% the size of all tumors. After two and a half years, all patients remained without recurrence and the cosmetic outcome was considered very good. Lu *et al.* [86] also evaluated the efficacy of three sessions of ALA-PDT after the excision of 32 BCCs without recurrence in one year of follow-up.

**Table 2:** Clinical studies of combined treatments with photodynamic therapy for squamous cell carcinoma.

MODEL	Photosensitizer (PDT)	Co-Adjuvant	Results	Side Effects	Reference
Patients: 13 BD	ALA	Surgery	100% complete response. No recurrence in 1 year	Moderate pain, mild local swelling, hyperpigmentation.	[86]
Patient: 1 BD	ALA	Imiquimod	No recurrence in 1 year	Pain, burning sensation, erythema, intermittent episodes of scaling and dryness.	[87]
Patients: 4 BD	ALA	Radiotherapy	100% complete response. no recurrence in 1 year	Improve radiotherapy side effects.	[89]
Patients: 22 BD	ALA	CO_2_ Laser	Combined therapy: 72.73% (8/11) complete remission, 1 recurrence (9%) after 1 month. CO_2_ Laser: 63.63% (7/11) complete remission, 5 recurrence (45.45%) after 6 months	Local side effects included mild to moderate edema, erosion, ulceration, delayed healing, prolonged pain, and scarring.	[90]
Patients: 5 SCC	ALA	Surgery	No recurrence in 6 months. 2 cases experienced recurrence in 1 year	Moderate pain, mild local swelling, hyperpigmentation.	[86]

**Table 3:** Clinical studies of combined treatments with photodynamic therapy for basal cell carcinoma.

Number of Tumors	Photosensitizer (PDT)	Co-Adjuvant	Results	Reference
10 BCCs and 8 SCCs	ALA	Surgery	4 BCC complete response. 14 lesions reduction in lesion area. 2 lesions increased. No recurrence after TFD + Surgery.	[103]
6 Morpheaform BCCs	MAL	Surgery	30%–50% reduction in volume after PDT. No recurrence in two years and a half.	[104]
32 BCCs	ALA	Surgery	No recurrence in one year.	[86]
1 nodular BCC	MAL	Imiquimod	50% reduction in volume after PDT. No recurrence 15 months.	[105]
3 giant BCCs	MAL	Imiquimod	20%–40% reduction in volume after the combined treatment. All needed surgery.	[43]
34 BCCs	ALA	Imiquimod	60% healing after PDT, and 75% after the combined one.	[106]
1 BCC	MAL	Imiquimod	No recurrence in two years	[107]
3 recurrent nodular BCCs on each patient (194 patients)	ALA	Er:YAG laser	Effectivity: 94.85% PDT, 91.75% laser, 98.9% after combined treatment.	[108]
75 BCCs	MAL	Er:YAG laser or diode laser	Effectivity: 81.2% PDT, 94.7% Er:YAG laser and 100% PDT diode laser.	[109]
13 nodular BCCs	MAL	CO_2_ laser	No recurrence. Mild hypopigmentation in 2 cases and mild discomfort with PDT.	[110]
56 nodular BCCs	MAL	Diode laser	Efficiency: 80.4% control group, 92.9% after combined treatment.	[111]
177 BCCs	MAL	CO_2_ laser	Efficiency: 97.1% with combined treatment. Mild hypopigmentation was occasionally seen and some discomfort with PDT.	[112]

BCC: basal cell carcinoma; ALA: aminolevulinic acid; MAL: methyl aminolevulinic acid; PDT: photodynamic therapy.

There are several papers about the combination of imiquimod and PDT for BCC. In one of them, 10 days after two sessions of PDT imiquimod was applied, five times a week for three weeks. Clinical clearing was obtained without recurrence over 15 months [105]. In another study, after three sessions of MAL-PDT, imiquimod was applied five times a week for six weeks. Three months later skin biopsies showed 22%–40% of lesion size reduction [44]. Osiecka *et al.* [106] treated 10 patients with ALA-PDT, and 24 patients with ALA-PDT plus imiquimod. Six patients (60%) were totally cured in the first group and four lesions (40%) decreased in size, whereas in the second group 18 lesions (75%) totally disappeared and six diminished. Requena *et al.* [107] reported a case of a giant recurrent basal cell carcinoma on the face successfully treated by a combination of MAL-PDT and imiquimod.

Regarding the combination with lasers, Smucler and Vlk studied the effectiveness of three different methods for the treatment of recurring nodular BCCs: ALA-PDT, Er:YAG laser, and the combination of both; this was the most effective, with an efficacy rate of 98.97% *vs.* 94.85% (PDT) and 91.75% (Er:YAG laser), and the best aesthetic results [108]. Later, the same group divided 67 patients into three groups based on tumor depth: <2 mm (PDT), 2–3 mm (Er:YAG laser ablation and PDT), and >3 mm (diode laser ablation and PDT). The treatment consisted of application of laser followed by MAL-PDT, repeated one and three weeks later. After six months, 100% clearance rate was observed in the group with the deepest tumors, 94.7% in that with tumors 2–3 mm in depth, and 81.2% in the group with the most superficial ones [109]. In other study, twelve patients were treated using combined CO_2_ laser and PDT, and after 18.1 months of follow-up no recurrences were present [110]. To evaluate the pretreatment with a fractional laser before ALA-PDT, Lippert *et al.* [111] ablated 56 nodular BCCs using a diode laser. Half of them were treated after three weeks using a fractional carbon dioxide laser, and the other 28 with curettage followed by MAL-PDT. Fifty-two BCCs of the laser group responded to MAL-PDT, compared with only 45 of 56 in the control group. Shokrollahi *et al.* [112] treated 110 patients with a total number of 177 lesions with combined therapy using a CO_2_ laser followed by two sessions of MAL-PDT one week apart. After a follow-up of 32.2 months the total recurrence-free rate was 97.1%. In 88.1% lesions, a single cycle of treatment was required, whereas 9.03% required two and three cycles in 0.56%. Recurrences were noted only in five cases (2.82%) and all of them were successfully retreated.

## 4. Conclusions

**Figure 3:**
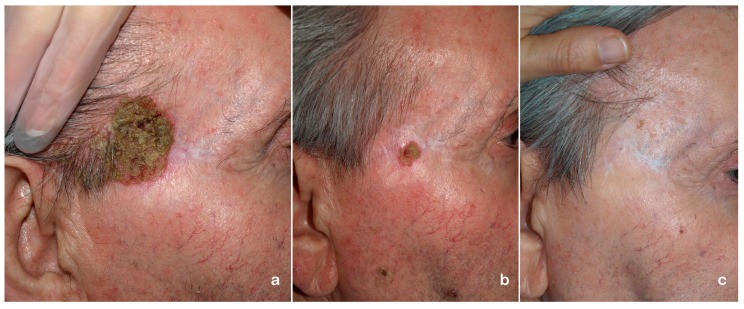
(**a**) Patient with recurrent Bowen disease after surgery and a cycle of methyl-aminolevulinate photodynamic therapy (MAL-PDT) (two sessions one week apart using 37 J·cm^−2^ Aktilite^®^ (Galderma SA, París, France); (**b**) Tumor persists one month after a second cycle of MAL-PDT and before treatment with topical imiquimod, five days per week during six weeks. (**c**) No recurrence after nine months of follow-up.

In general, the combination with other treatments improves the results of PDT. The possibility of adding two different mechanisms of action seems to be a good strategy to improve results and overcome PDT limitations (Figure 3). However, most of the performed studies include a small number of patients, the results are only clinically evaluated without histological confirmation, and finally the time of follow-up is not very long. A better knowledge of the molecular mechanisms implicated in PDT for the different NMSC will lead us not only to understand better which are the most promising combinations but also to explore new strategies to optimize them.

## References

[1] Zhao B., He Y.Y. (2010). Recent advances in the prevention and treatment of skin cancer using photodynamic therapy. Expert Rev. Anticancer Ther..

[2] Katalinic A., Kunze U., Schafer T. (2003). Epidemiology of cutaneous melanoma and non-melanoma skin cancer in Schleswig-Holstein, Germany: Incidence, clinical subtypes, tumour stages and localization (epidemiology of skin cancer). Br. J. Dermatol..

[3] Eisemann N., Waldmann A., Geller A.C., Weinstock M.A., Volkmer B., Greinert R., Breitbart E.W., Katalinic A. (2014). Non-melanoma skin cancer incidence and impact of skin cancer screening on incidence. J. Investig. Dermatol..

[4] Sidoroff M.D., Thaler P. (2010). Taking treatment decisions in non-melanoma skin cancer—The place for topical photodynamic therapy (PDT). Photodiagn. Photodyn. Ther..

[5] Kim Y., He Y.Y. (2014). Ultraviolet radiation-induced non-melanoma skin cancer: Regulation of DNA damage repair and inflammation. Genes Dis..

[6] Ericson M.B., Wennberg A.M., Larkö O. (2008). Review of photodynamic therapy in actinic keratosis and basal cell carcinoma. Ther. Clin. Risk Manag..

[7] Koyuncuer A. (2014). Histopathological evaluation of non-melanoma skin cancer. World J. Surg. Oncol..

[8] Berking C., Hauschild A., Kölbl O., Mast G., Gutzmer R. (2014). Basal cell carcinoma-treatments for the commonest skin cancer. Dtsch. Arztebl. Int..

[9] Blanplain C., Fuchs E. (2014). Stem cell plasticity. Plasticity of epithelial stem cell in tissue regeneration. Science.

[10] Dlugosz A., Merlino G., Yuspa S.H. (2002). Progress in cutaneous cancer research. J. Investig. Dermatol. Symp. Proc..

[11] Goldman G.D. (1998). Squamous cell cancer: A practical approach. Semin. Cutan. Med. Surg..

[12] Kolk A., Wolff K.D., Smeets R., Kesting M., Hein R., Eckert A.W. (2014). Melanotic and non-melanotic malignancies of the face and external ear—A review of current treatment concepts and future options. Cancer Treat. Rev..

[13] Martorell-Calatayud A., Sanmartín O., Cruz J., Guillén C. (2013). Cutaneous squamous cell carcinoma: Defining the high-risk variant. Actas Dermosifiliogr..

[14] Hofbauer G.F., Bouwes J.N., Euvrard S. (2010). Organ transplantation and skin cancer: Basic problems and new perspectives. Exp. Dermatol..

[15] Cockerell C.J. (2000). Histopathology of incipient intraepidermal squamous cell carcinoma (“actinic keratosis”). J. Am. Acad. Dermatol..

[16] Fernández-Figueras M.T., Carrato C., Sáenz X., Puig L., Musulen E., Ferrándiz C., Ariza A. (2015). Actinic keratosis with atypical basal cells (AK I) is the most common lesion associated with invasive squamous cell carcinoma of the skin. J. Eur. Acad. Dermatol. Venereol..

[17] Patel R.V., Frankel A., Goldenberg G. (2011). An update on nonmelanoma skin cancer. J. Clin. Aesthet. Dermatol..

[18] Kauvar A.N., Cronin T., Roenigk R., Hruza G., Bennett R. (2015). Consensus for nonmelanoma skin cancer treatment: Basal cell carcinoma, including a cost analysis of treatment methods. Dermatol. Surg..

[19] Ferrándiz C., Fonseca-Capdevila E., García-Diez A., Guillén-Barona C., Belinchón-Romero I., Redondo-Bellón P., Moreno-Giménez J.C., Senán R. (2014). Spanish adaptation of the European guidelines for the evaluation and treatment of actinic keratosis. Actas Dermosifiliogr..

[20] Morton C.A. (2004). Photodynamic therapy for nonmelanoma skin cancer—And more?. Arch. Dermatol..

[21] Marmur E.S., Schmults C.D., Goldberg D.J. (2004). A review of laser and photodynamic therapy for the treatment of nonmelanoma skin cancer. Dermatol. Surg..

[22] Kormeili T., Yamauchi P.S., Lowe N.J. (2004). Topical photodynamic therapy in clinical dermatology. Br. J. Dermatol..

[23] Lehmann P. (2007). Methyl aminolaevulinate-photodynamic therapy: A review of clinical trials in the treatment of actinic keratoses and nonmelanoma skin cancer. Br. J. Dermatol..

[24] Braathen L.R., Szeimies R.M., Basset-Seguin N., Bissonnette R., Foley P., Pariser D., Roelandts R., Wennberg A.M., Morton C.A. (2007). Guidelines on the use of photodynamic therapy for nonmelanoma skin cancer: An international consensus. J. Am. Acad. Dermatol..

[25] Steinbauer J.M., Schreml S., Kohl E.A., Karrer S., Landthaler M., Szeimies R.M. (2010). Photodynamic therapy in dermatology. J. Dtsch. Dermatol. Ges..

[26] Morton C., Szeimies R.M., Sidoroff A., Wennberg A.M., Basset-Seguin N., Calzavara-Pinton P., Gilaberte Y., Hofbauer G., Hunger R., Karrer S. (2015). European Dermatology Forum Guidelines on topical photodynamic therapy. Eur. J. Dermatol..

[27] Choudhary S., Nouri K., Elsaie M.L. (2009). Photodynamic therapy in dermatology: A review. Lasers Med. Sci..

[28] Christensen E., Warloe T., Kroon S., Funk J., Helsing P., Soler A.M., Stang H.J., Vatne O., Mørk C. (2010). Guidelines for practical use of MAL-PDT in non-melanoma skin cancer. J. Eur. Acad. Dermatol. Venereol..

[29] Henderson B.W., Dougherty T.J. (1992). How does photodynamic therapy work?. Photochem. Photobiol..

[30] Mfouo-Tynga I., Abrahamse H. (2015). Cell death pathways and phthalocyanine as an efficient agent for photodynamic cancer therapy. Int. J. Mol. Sci..

[31] Blume J.E., Oseroff A.R. (2007). Aminolevulinic acid photodynamic therapy for skin cancers. Dermatol. Clin..

[32] Evangelou G., Farrar M.D., Cotterell L., Andrew S., Tosca A.D., Watson R.E., Rhodes L.E. (2012). Topical photodynamic therapy significantly reduces epidermal Langerhans cells during clinical treatment of basal cell carcinoma. Br. J. Dermatol..

[33] Morton C.A., Szeimies R.M., Sidoroff A, Braathen L.R. (2013). European guidelines for topical photodynamic therapy part 1: Treatment delivery and current indications-actinic keratoses, Bowen’s disease, basal cell carcinoma. J. Eur. Acad. Dermatol. Venereol..

[34] Kasche A., Luderschmidt S., Ring J., Hein R. (2006). Photodynamic therapy induces less pain in patients treated with methyl aminolevulinate compared to aminolevulinic acid. J. Drugs Dermatol..

[35] Halldin C.B., Gillstedt M., Paoli J., Wennberg A.M., Gonzalez H. (2011). Predictors of pain associated with photodynamic therapy: A retrospective study of 658 treatments. Acta Derm. Venereol..

[36] Attili S.K., Dawe R., Ibbotson S. (2011). A review of pain experienced during topical photodynamic therapy-our experience in Dundee. Photodiagn. Photodyn. Ther..

[37] Gholam P., Denk K., Sehr T., Enk A., Hartmann M. (2010). Factors influencing pain intensity during topical photodynamic therapy of complete cosmetic units for actinic keratoses. J. Am. Acad. Dermatol..

[38] Borroni R.G., Carugno A., Rivetti N., Arbustini E., Brazzelli V. (2013). Risk of acute postoperative hypertension after topical photodynamic therapy for non-melanoma skin cancer. Photodermatol. Photoimmunol. Photomed..

[39] McKay K.M., Sambrano B.L., Fox P.S., Bassett R.L., Chon S., Prieto V.G. (2013). Thickness of superficial basal cell carcinoma (sBCC) predicts imiquimod efficacy: A proposal for a thickness-based definition of sBCC. Br. J. Dermatol..

[40] Roozeboom M.H., van Kleef L., Arits A.H., Mosterd K., Winnepenninckx V.J., van Marion A.M., Nelemans P.J., Kelleners-Smeets N.W. (2015). Tumor thickness and adnexal extension of superficial basal cell carcinoma (sBCC) as determinants of treatment failure for methyl aminolevulinate (MAL)-photodynamic therapy (PDT), imiquimod, and 5-fluorouracil (FU). J. Am. Acad. Dermatol..

[41] Christensen E., Mørk C., Foss O.A. (2011). Pre-treatment deep curettage can significantly reduce tumour thickness in thick Basal cell carcinoma while maintaining a favourable cosmetic outcome when used in combination with topical photodynamic therapy. J. Skin Cancer.

[42] Ramirez D.P., Kurachi C., Inada N.M., Moriyama L.T., Salvio A.G., Vollet Filho J.D., Pires L., Buzzá H.H., de Andrade C.T., Greco C. (2014). Experience and BCC subtypes as determinants of MAL-PDT response: Preliminary results of a national Brazilian project. Photodiagn. Photodyn. Ther..

[43] Madan V., West C.A., Murphy J.V., Lear J.T. (2009). Sequential treatment of giant basal cell carcinomas. J. Plast. Reconstr. Aesthet. Surg..

[44] Fritsch C., Goerz G., Ruzicka T. (1998). Photodynamic therapy in dermatology. Arch. Dermatol..

[45] Horn M., Wolf P., Wulf H.C., Warloe T., Fritsch C., Rhodes L.E., Kaufmann R., de Rie M., Legat F.J., Stender I.M. (2003). Topical methyl aminolaevulinate photodynamic therapy in patients with basal cell carcinoma prone to complications and poor cosmetic out-come with conventional treatment. Br. J. Dermatol..

[46] Rhodes L.E., de Rie M.A., Leifsdottir R., Yu R.C., Bachmann I., Goulden V., Wong G.A., Richard M.A., Anstey A., Wolf P. (2007). Five-year follow-up of a randomized, prospective trial of topical methyl aminolevulinate photodynamic therapy *vs* surgery for nodular basal cell carcinoma. Arch. Dermatol..

[47] Szeimies R.M., Ibbotson S., Murrell D.F., Rubel D., Frambach Y., de Berker D., Dummer R., Kerrouche N., Villemagne H. (2008). A clinical study comparing methyl aminolevulinate photodynamic therapy and surgery in small superficial basal cell carcinoma (8–20 mm), with a 12-month follow-up. J. Eur. Acad. Dermatol. Venereol..

[48] Vinciullo C., Elliott T., Francis D., Gebauer K., Spelman L., Nguyen R., Weightman W., Sheridan A., Reid C., Czarnecki D. (2005). Photodynamic therapy with topical methyl aminolaevulinate for “difficult-to-treat” basal cell carcinoma. Br. J. Dermatol..

[49] Morton C.A., Whitehurst C., McColl J.H., Moore J.V., MacKie R.M. (2001). Photodynamic therapy for large or multiple patches of bowen disease and basal cell carcinoma. Arch. Dermatol..

[50] Fantini F., Greco A., del Giovane C., Cesinaro A.M., Venturini M., Zane C., Surrenti T., Peris K., Calzavara-Pinton P.G. (2011). Photodynamic therapy for basal cell carcinoma: Clinical and pathological determinants of response. J. Eur. Acad. Dermatol. Venereol..

[51] Lopez N., Meyer-Gonzalez T., Herrera-Acosta E., Bosch R., Castillo R., Herrera E. (2012). Photodynamic therapy in the treatment of extensive Bowen’s disease. J. Dermatol. Treat..

[52] Stables G.I., Stringer M.R., Robinson D.J., Ash D.V. (1997). Large patches of Bowen’s disease treated by topical aminolaevulinic acid photodynamic therapy. Br. J. Dermatol..

[53] Calzavara-Pinton P.G., Venturini M., Sala R., Capezzera R., Parrinello G., Specchia C., Zane C. (2008). Methyl aminolevulinate-based photodynamic therapy of Bowen’s disease and squamous cell carcinoma. Br. J. Dermatol..

[54] Park J.Y., Kim S.K., Cho K.H., Kim Y.C. (2013). Huge Bowen’s disease: A pitfall of topical photodynamic therapy. Photodiagn. Photodyn. Ther..

[55] Morton C.A., Campbell S., Gupta G., Keohane S., Lear J., Zaki I., Walton S., Kerrouche N., Thomas G., Soto P. (2006). Intraindividual, right-left comparison of topical methyl aminolaevulinate-photodynamic therapy and cryotherapy in subjects with actinic keratoses: A multicentre, randomized controlled study. Br. J. Dermatol..

[56] Kaufmann R., Spelman L., Weightman W., Reifenberger J., Szeimies R.M., Verhaeghe E., Kerrouche N., Sorba V., Villemagne H., Rhodes L.E. (2008). Multicentre intraindividual randomized trial of topical methyl aminolaevulinate-photodynamic therapy *vs.* cryotherapy for multiple actinic keratoses on the extremities. Br. J. Dermatol..

[57] Morton C.A., Birnie A.J., Eedy D.J. (2014). British Association of Dermatologists’ guidelines for the management of squamous cell carcinoma *in situ* (Bowen’s disease) 2014. Br. J. Dermatol..

[58] Nissen C.V., Philipsen P.A., Wulf H.C. (2015). PpIX formation after topical application of methyl aminolevulinate and BF-200 ALA declines with age. Br. J. Dermatol..

[59] Tehranchinia Z., Rahimi H., Ahadi M.S., Ahadi M.S. (2013). Aminolevulinic Acid-photodynamic therapy of Basal cell carcinoma and factors affecting the response to treatment: A clinical trial. Indian J. Dermatol..

[60] Holohan C., van Schaeybroeck S., Longley D.B., Johnston P.G. (2013). Cancer drug resistance: An evolving paradigm. Nat. Rev. Cancer.

[61] Longley D.B., Johnston P.G. (2005). Molecular mechanisms of drug resistance. J. Pathol..

[62] Gillet J.P., Gottesman M. (2010). Mechanisms of multidrug resistance in cancer. Methods Mol. Biol..

[63] Hombach-Klonisch S., Natarajan S., Thanasupawat T., Medapati M., Pathak A., Ghavami S., Klonisch T. (2014). Mechanisms of therapeutic resistance in cancer (stem) cells with emphasis on thyroid cancer cells. Front. Endocrinol..

[64] Al-Dimassi S., Abou-Antoun T., El-Sibai M. (2014). Cancer cell resistance mechanisms: A mini review. Clin. Transl. Oncol..

[65] Maydan E., Nooothet P.K., Goldman M.P. (2006). Case reports: Development of a keratoacanthoma after topical photodynamic therapy with 5-aminolevolunic acid. J. Drugs Dermatol..

[66] Fiechter S., Skaria A., Nievergelt H., Anex R., Borradori L., Parmentier L. (2012). Facial basal cell carcinomas recurring after photodynamic therapy: A retrospective analysis of histological subtypes. Dermatology.

[67] Bardazzi F., Loi C., Magnano M., Burtica E.C., Giordano F., Patrizi A. (2015). Methyl-aminolevulinic acid photodynamic therapy for actinic keratoses: An useful treatment or a risk factor? A retrospective study. J. Dermatol. Treat..

[68] Kessel D. (2015). More Adventures in Photodynamic Therapy. Int. J. Mol. Sci..

[69] Agostinis P., Berg K., Cengel K.A., Foster T.H., Girotti A.W., Gollnick S.O., Hahn S.M., Hamblin M.R., Juzeniene A., Kessel D. (2011). Photodynamic therapy of cancer: An update. CA Cancer J. Clin..

[70] Casas A., di Venosa G., Hasan T., Batlle A. (2011). Mechanisms of resistance to photodynamic therapy. Curr. Med. Chem..

[71] Muthusamy V., Piva T.J. (2010). The UV response of the skin: A review of the MAPK, NF-κB and TNF-α signal transduction pathways. Arch. Dermatol. Res..

[72] Kuźbicki Ł., Lange D., Stanek-Widera A., Chwirot B.W. (2011). Different expression of cyclooxygenase-2 (COX-2) in selected nonmelanocytic human cutaneous lesions. Folia Histochem. Cytobiol..

[73] Müller-Decker K. (2011). Cyclooxygenase-dependent signaling is causally linked to non-melanoma skin carcinogenesis: Pharmacological, genetic, and clinical evidence. Cancer Metastasis Rev..

[74] Walls B., Jordan L., Diaz L., Miller R. (2014). Targeted therapy for cutaneous oncology: A review of novel treatment options for non-melanoma skin cancer: Part I. J. Drugs Dermatol..

[75] Bahner J.D., Bordeaux J.S. (2013). Non-melanoma skin cancers: Photodynamic therapy, cryotherapy, 5-fluorouracil, imiquimod, diclofenac, or what? Facts and controversies. Clin. Dermatol..

[76] Gilbert D.J. (2005). Treatment of actinic keratoses with sequential combination of 5-fluorouracil and photodynamic therapy. J. Drugs Dermatol..

[77] Martin G. (2011). Prospective, case-based assessment of sequential therapy with topical Fluorouracil cream 0.5% and ALA-PDT for the treatment of actinic keratosis. J. Drugs Dermatol..

[78] Shaffelburg M. (2009). Treatment of actinic keratoses with sequential use of photodynamic therapy; and imiquimod 5% cream. J. Drugs Dermatol..

[79] Serra-Guillén C., Nagore E., Hueso L., Traves V., Messeguer F., Sanmartín O., Llombart B., Requena C., Botella-Estrada R., Guillén C. (2012). A randomized pilot comparative study of topical methyl aminolevulinate photodynamic therapy *vs.* imiquimod 5% *vs.* sequential application of both therapies in immunocompetent patients with actinic keratosis: Clinical and histologic outcomes. J. Am. Acad. Dermatol..

[80] Held L., Eigentler T.K., Leiter U., Garbe C., Berneburg M.J. (2013). Effective combination of photodynamic therapy and imiquimod 5% cream in the treatment of actinic keratoses: Three cases. Biomed. Res. Int..

[81] Akita Y., Kozaki K., Nakagawa A., Saito T., Ito S., Tamada Y., Fujiwara S., Nishikawa N., Uchida K., Yoshikawa K. (2004). Cyclooxygenase-2 is a possible target of treatment approach in conjunction with photodynamic therapy for various disorders in skin and oral cavity. Br. J. Dermatol..

[82] Bagazgoitia L., Cuevas J., Juarranz A., Jaén P. (2011). Photodynamic therapy reduces the histological features of actinic damage and the expression of early oncogenic markers. Br. J. Dermatol..

[83] Van der Geer S., Krekels G.A. (2009). Treatment of actinic keratoses on the dorsum of the hands: ALA-PDT *vs.* diclofenac 3% gel followed by ALA-PDT. A placebo-controlled, double-blind, pilot study. J. Dermatol. Treat..

[84] Berman B., Nestor M.S., Newburger J., Park H., Swenson N. (2014). Treatment of facial actinic keratoses with aminolevulinic acid photodynamic therapy (ALA-PDT) or ingenol mebutate 0.015% gel with and without prior treatment with ALA-PDT. J. Drugs Dermatol..

[85] Togsverd-Bo K., Lei U., Erlendsson A.M., Taudorf E.H., Philipsen P.A., Wulf H.C., Skov L., Hædersdal M. (2015). Combination of ablative fractional laser and daylight-mediated photodynamic therapy for actinic keratosis in organ transplant recipients—A randomized controlled trial. Br. J. Dermatol..

[86] Lu Y.G., Wang Y.Y., Yang Y.D., Zhang X.C., Gao Y., Yang Y., Zhang J.B., Li G.L. (2014). Efficacy of topical ALA-PDT combined with excision in the treatment of skin malignant tumor. Photodiagn. Photodyn. Ther..

[87] Sotiriou E., Lallas A., Apalla Z., Ioannides D. (2011). Treatment of giant Bowen’s disease with sequential use of photodynamic therapy and imiquimod cream. Photodermatol. Photoimmunol. Photomed..

[88] Rong Y., Zuo L., Shang L., Bazan J.G. (2015). Radiotherapy treatment for nonmelanoma skin cancer. Expert Rev. Anticancer Ther..

[89] Nakano A., Watanabe D., Akita Y., Kawamura T., Tamada Y., Matsumoto Y. (2011). Treatment efficiency of combining photodynamic therapy and ionizing radiation for Bowen’s disease. J. Eur. Acad. Dermatol. Venereol..

[90] Cai H., Wang Y.X., Zheng J.C., Sun P., Yang Z.Y., Li Y.L., Liu X.Y., Li Q., Liu W. (2015). Photodynamic therapy in combination with CO_2_ laser for the treatment of Bowen’s disease. Lasers Med. Sci..

[91] Ishida N., Watanabe D., Akita Y., Nakano A., Yamashita N., Kuhara T., Yanagishita T., Takeo T., Tamada Y., Matsumoto Y. (2009). Etretinate enhances the susceptibility of human skin squamous cell carcinoma cells to 5-aminolaevulic acid-based photodynamic therapy. Clin. Exp. Dermatol..

[92] Anand S., Honari G., Hasan T., Elson P., Maytin E.V. (2009). Low-dose methotrexate enhances aminolevulinate-based photodynamic therapy in skin carcinoma cells *in vitro* and *in vivo*. Clin. Cancer Res..

[93] Caini S., Boniol M., Tosti G., Magi S., Medri M., Stanganelli I., Palli D., Assedi M., Marmol V.D., Gandini S. (2014). Vitamin D and melanoma and non-melanoma skin cancer risk and prognosis: A comprehensive review and meta-analysis. Eur. J. Cancer.

[94] Pereira F., Larriba M.J., Muñoz A. (2012). Vitamin D and colon cancer. Endocr. Relat. Cancer.

[95] Uhmann A., Niemann H., Lammering B., Henkel C., Hess I., Nitzki F., Fritsch A., Prüfer N., Rosenberger A., Dullin C. (2011). Antitumoral effects of calcitriol in basal cell carcinomas involve inhibition of hedgehog signaling and induction of vitamin D receptor signaling and differentiation. Mol. Cancer Ther..

[96] Cicarma E., Tuorkey M., Juzeniene A., Ma L.W., Moan J. (2009). Calcitriol treatment improves methyl aminolaevulinate-based photodynamic therapy in human squamous cell carcinoma A431 cells. Br. J. Dermatol..

[97] Anand S., Wilson C., Hasan T., Maytin E.V. (2011). Vitamin D3 enhances the apoptotic response of epithelial tumors to aminolevulinate-based photodynamic therapy. Cancer Res..

[98] Anand S., Rollakanti K.R., Horst R.L., Hasan T., Maytin E.V. (2014). Combination of oral vitamin D3 with photodynamic therapy enhances tumor cell death in a murine model of cutaneous squamous cell carcinoma. Photochem. Photobiol..

[99] Rollakanti K., Anand S., Maytin E.V. (2015). Topical calcitriol prior to photodynamic therapy enhances treatment efficacy in non-melanoma skin cancer mouse models. Proc. SPIE Int. Soc. Opt. Eng..

[100] Gilaberte Y., Milla L., Salazar N., Vera-Alvarez J., Kourani O., Damian A., Rivarola V., Roca M.J., Espada J., González S. (2014). Cellular intrinsic factors involved in the resistance of squamous cell carcinoma to photodynamic therapy. J. Investig. Dermatol..

[101] Martinez-Carpio P.A., Trelles M.A. (2010). The role of epidermal growth receptor in photodynamic therapy: A review of the literature and proposal for future investigations. Lasers Med. Sci..

[102] Weyergang A., Selbo P.K., Berg K. (2013). Sustained ERK inhibition by EGFR targeting therapies is a predictive factor for synergistic cytotoxicity with PDT as neoadjuvant therapy. Biochim. Biophys. Acta.

[103] Jeremic G., Brandt M.G., Jordan K., Doyle P.C., Yu E., Moore C.C. (2011). Using photodynamic therapy as a neoadjuvant treatment in the surgical excision of nonmelanotic skin cancers: Prospective study. J. Otolaryngol. Head Neck Surg..

[104] Torres T., Fernandes I., Costa V., Selores M. (2011). Photodynamic therapy as adjunctive therapy for morpheaform basal cell carcinoma. Acta Dermatovenerol. Alp. Pannonica Adriat..

[105] Devirgiliis V., Panasiti V., Curzio M., Gobbi S., Rossi M., Roberti V., Calvieri S. (2008). Complete remission of nodular basal cell carcinoma after combined treatment with photodynamic therapy and imiquimod 5% cream. Dermatol. Online J..

[106] Osiecka B., Jurczyszyn K., Ziółkowski P. (2012). The application of Levulan-based photodynamic therapy with imiquimod in the treatment of recurrent basal cell carcinoma. Med. Sci. Monit..

[107] Requena C., Messeguer F., Llombart B., Serra-Guillén C., Guillén C. (2012). Facial extensive recurrent basal cell carcinoma: Successful treatment with photodynamic therapy and imiquimod 5% cream. Int. J. Dermatol..

[108] Smucler R., Vlk M. (2008). Combination of Er:YAG laser and photodynamic therapy in the treatment of nodular basal cell carcinoma. Lasers Surg. Med..

[109] Smucler R., Kriz M., Lippert J., Vlk M. (2012). Ultrasound guided ablative-laser assisted photodynamic therapy of basal cell carcinoma (US-aL-PDT). Photomed. Laser Surg..

[110] Whitaker I.S., Shokrollahi K., James W., Mishra A., Lohana P., Murison M.C. (2007). Combined CO_2_ laser with photodynamic therapy for the treatment of nodular basal cell carcinomas. Ann. Plast. Surg..

[111] Lippert J., Smucler R., Vlk M. (2013). Fractional carbon dioxide laser improves nodular basal cell carcinoma treatment with photodynamic therapy with methyl 5-aminolevulinate. Dermatol. Surg..

[112] Shokrollahi K., Javed M., Aeuyung K., Ghattaura A., Whitaker I.S., O’Leary B., James W., Murison M. (2014). Combined carbon dioxide laser with photodynamic therapy for nodular and superficial basal cell carcinoma. Ann. Plast. Surg..

